# Enhanced acarbose production by *Streptomyces* M37 using a two-stage fermentation strategy

**DOI:** 10.1371/journal.pone.0166985

**Published:** 2017-02-24

**Authors:** Fei Ren, Long Chen, Shuangli Xiong, Qunyi Tong

**Affiliations:** 1 School of Life Science and Technology, Southwest University of Science and Technology, Mianyang, Sichuan, China; 2 State Key Laboratory of Food Science and Technology, Jiangnan University, Wuxi, Jiangsu, China; 3 Engineering Research Center for Biomass Resource Utillization and Modification of Sichuan Province, Mianyang, Sichuan, China; Texas A&M University College Station, UNITED STATES

## Abstract

In this work, we investigated the effect of pH on *Streptomyces* M37 growth and its acarbose biosynthesis ability. We observed that low pH was beneficial for cell growth, whereas high pH favored acarbose synthesis. Moreover, addition of glucose and maltose to the fermentation medium after 72 h of cultivation promoted acarbose production. Based on these results, a two-stage fermentation strategy was developed to improve acarbose production. Accordingly, pH was kept at 7.0 during the first 72 h and switched to 8.0 after that. At the same time, glucose and maltose were fed to increase acarbose accumulation. With this strategy, we achieved an acarbose titer of 6210 mg/L, representing an 85.7% increase over traditional batch fermentation without pH control. Finally, we determined that the increased acarbose production was related to the high activity of glutamate dehydrogenase and glucose 6-phosphate dehydrogenase.

## Introduction

Acarbose (acarviosyl-1,4-maltose) is a competitive α-glucosidase inhibitor [1] widely used in the treatment of non-insulin-dependent diabetes mellitus (NIDDM) [2,3]. Acarbose is a complex oligosaccharide known to reduce and slow down the intestinal absorption of glucose. As a result, it lowers the postprandial increase in blood glucose levels in NIDDM patients. Its medical effect is based on a decreased release of glucose from starch- and sucrose-containing foods in the human intestine, which leads to reduced levels of blood glucose and serum insulin [4]. Acarbose was identified in strains of the genera *Actinoplanes* and *Streptomyces* [5,6]. It was also reported to belong to a mixed-growth-associated type of secondary metabolites; its biosynthesis being independent of cell growth [7].

**Fig 1:**
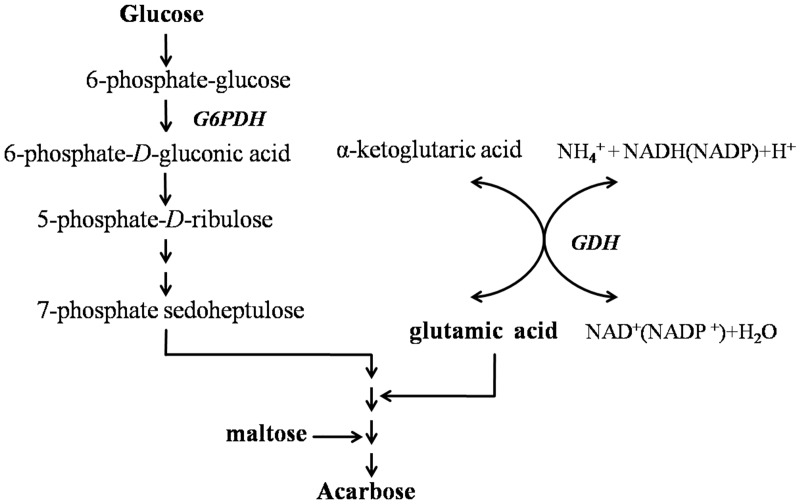
Postulated acarbose biosynthetic pathway. Glucose and maltose are the carbon sources for acarbose biosynthesis.

Structurally, acarbose consists of moieties of acarviose and maltose. Work on acarbose biosynthesis has demonstrated that glucose is converted to glucose-6-phosphate, which is then metabolized to sedoheptulose 7-phosphate via the hexose monophosphate (HMP) pathway, subsequently forming valienamine [8]. Glutamate was reported to be the primary source of N-glycosidic bonds in acarbose [9]. The maltosyl unit of acarbose is derived directly from maltose [10]. The biosynthetic pathway of acarbose is shown in Fig 1.

Medium pH is one of the most important environmental parameters affecting cell growth and product formation. Controlling medium pH is a common way to optimize metabolite and enzyme production. Although previous studies have investigated various physiological factors that influence acarbose production [11–14], the effect of medium pH on acarbose biosynthesis has not been fully explored. The aim of the current investigation was to examine the effect of medium pH on acarbose production. Furthermore, we also investigated the effect of feeding glucose and maltose to promote acarbose production. Finally, we propose a two-stage fermentation strategy to improve acarbose production by *Streptomyces* M37.

## Materials and methods

### Microorganism

*Streptomyces* M37 (GenBank KJ888155), a high acarbose-producing strain, was used in this study. The strain was isolated and mutated in our previous study [15]. Spores of *Streptomyces* M37 were stored at -80°C in 25% (v/v) sterile glycerol. Prior to the study, *Streptomyces* M37 was activated on agar plates at 28°C for about 5 days until visible white colonies emerged.

### Culture media

Agar plate medium was composed of (g/L): sucrose, 20; K_2_HPO_4_·3H_2_O, 1.0; FeSO_4_·7H_2_O, 0.02; MgSO_4_, 1.0; NaNO_3_, 2.0; peptone, 1.0; and agar, 20. Unless otherwise stated, the initial pH of the medium was adjusted to 7.0 with 2 M NaOH and then sterilized by steam autoclaving at 121°C for 20 min.

The seed culture for fermentation inoculation was prepared in a medium consisting of (g/L): soluble starch, 15; sucrose, 40; NH_4_Cl, 3.0; peptone, 4.0; yeast extract, 2.0; K_2_HPO_4_·3H_2_O, 1.5; FeSO_4_·7H_2_O, 0.02; MgSO_4_, 1.0; and CaCO_3_, 3.0.

The fermentation medium contained (g/L): sucrose, 40; glucose, 15; maltose, 10; peptone, 2.0; yeast extract, 1.0; K_2_HPO_4_·3H_2_O, 1.5; NH_4_Cl, 6.0; MgSO_4_, 1.0; and FeSO_4_·7H_2_O, 0.02. Glucose and maltose were sterilized separately to prevent Maillard reactions and added to the medium before inoculation. Unless otherwise stated, the feeding medium was composed of (g/L): glucose, 134; and maltose, 266.

### Culture conditions

A loop of *Streptomyces* M37 spores was inoculated onto an agar plate and incubated at 28°C in an electro-thermal incubator. After 5 days of incubation, seed cultures were prepared by inoculating 1-cm^2^ mycelia into 250-mL shake-flasks containing 100 mL fresh medium. Cultivation was performed for 3 days at 28°C with stirring at 200 rpm.

The effect of initial pH on acarbose fermentation was assessed in 250-mL Erlenmeyer flasks containing 90 mL medium shaken at 200 rpm. The effect of pH (constant pH control or two-stage pH control strategy) on acarbose fermentation was studied in a 5-L fermentor (KoBioTech Co., Ltd., Incheon, Korea) with a working volume of 3.0 L. A 10% (v/v) seed culture was inoculated into the fermentor. Aeration rate was kept at 1.0 L/L/min and agitation speed was set to 200 rpm. Procedures for the two-stage fermentation strategy were carried out as follows. In the first stage (72 h), pH was kept at 7.0; in the second stage, pH was maintained at 8.0 using 2 M NaOH and 2 M H_2_SO_4_, while precursors for acarbose biosynthesis (glucose and maltose) were added to the fermentation broth. All fermentations were carried out at 28°C for 7 days.

### Analytical methods

Mycelia were harvested by centrifugation (10,000 rpm, 10 min) and then dried at 85°C in an oven to obtain a constant dry cell weight (DCW). The resulting supernatants were transferred to sterile microcentrifuge tubes and stored at -20°C until analysis. Acarbose titer was measured by high performance liquid chromatography according to Li et al. [11]. The pH was measured using a digital pH meter (PHS-3C; Mettler Toledo, Columbus, OH, USA). Protein content was measured by the Bradford method using bovine serum albumin as a standard [16]. The concentration of reducing sugars (glucose and maltose) in the broth was measured by the dinitrosalicylic method [17]. Glutamate dehydrogenase (GDH) activity was measured spectrophotometrically at 30°C by monitoring the oxidation of NADH or NADPH at 340 nm [15]. Glucose 6-phosphate dehydrogenase (G6PDH) activity was determined according to the method described by Ruijter et al. [18].

### Statistical analysis

Data are expressed as means of triplicate determinations, unless otherwise stated. Statistical significance was assessed with one-way analysis of variance (ANOVA) using SPSS 20.0 software (SPSS Inc., Chicago, IL, USA) for Windows^®^. A *p* value < 0.05 was considered to be statistically significant throughout the study.

## Results and discussion

### Effect of initial pH on cell growth and acarbose production during batch fermentation

To study the effect of pH on *Streptomyces* M37 growth and acarbose production, we performed cultivations in 250-mL Erlenmeyer flasks containing 100 mL fermentation medium for a period of 168 h. The initial pH ranged from 6.5 to 8.5. As shown in Table 1, the highest DCW (21.58 g/L) was achieved at an initial pH of 7.0. DCW decreased as culture medium pH increased from 7.0 to 8.0, indicating that an initial pH higher than 7.0 was not beneficial for cell growth. Maximum specific acarbose production (173.7 mg/L DCW) was obtained at an initial pH of 8.0. This was 21.3%, 14.5%, 9.7%, and 16.9% higher than that at pH 6.5, 7.0, 7.5, and 8.5, respectively. At 3345 mg/L, acarbose production was higher at an initial pH of 8.0, than at other initial pH values. At the same time, specific acarbose production (C_max_(mg/L DCW)) increased along with initial pH (in the 6.5 to 8.0 range). It should be noted that in all experiments the final pH after 168 h of cultivation was lower than 6.0 (Table 1).

**Table 1:** Effect of culture medium initial pH on cell growth and acarbose yield during flask fermentation.

Initial pH	6.5		7.0	7.5	8.0	8.5
Final pH	5.58 ± 0.26		5.53 ± 0.24	5.59 ± 0.22	5.61 ± 0.27	5.64 ± 0.25
Max DCW (g/L)	20.20 ± 1.02		21.58 ± 1.05	18.43 ± 0.88	17.53 ± 0.84	15.36 ± 0.77
P_max_ (mg/L)	3206 ± 161		3177 ± 157	3223 ± 149	3345 ± 146	2470 ± 130
C_max_(mg/g DCW)		143.2 ± 8.7	151.7 ± 10.1	158.4 ±12.6	173.7 ± 12.4	148.6 ± 11.8

Each value represents the mean of three separate determinations ± standard deviation.

DCW = Dry cell weight.

Results demonstrated that the relatively low initial pH was suitable for cell growth, whereas a higher pH favored acarbose biosynthesis. Thus, the optimal pH for *Streptomyces* M37 growth differed from that for target metabolite production. A similar result was observed in the case of propionic acid fermentation [19].

### Effect of controlling pH on acarbose production in a 5-L fermentor

**Fig 2:**
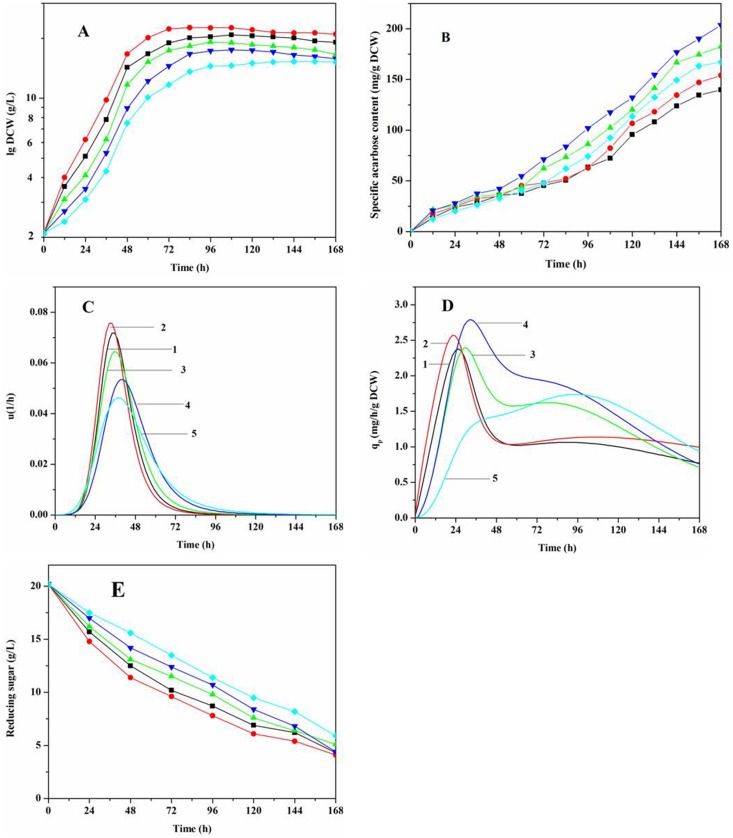
Effect of pH on *Streptomyces* M37 fermentation. (A) DCW, (B) specific acarbose content, (C) specific growth rate (μ), (D) specific acarbose production rate (*q*_p_), and (E) reducing sugars (glucose and maltose). Experiments were carried out in a 5-L fermentor. NaOH (2 M) and H_2_SO_4_ (2 M) were used to buffer the pH. ■ curve 1, pH 6.5; ● curve 2, pH 7.0; ▲ curve 3, pH 7.5; ▼ curve 4, pH 8.0; and ◆ curve 5, pH 8.5. Data correspond to mean ± S.D. (*n* = 3). Error bars were omitted for clarity.

The effect of pH on acarbose batch fermentation was further investigated by controlling the pH of the medium (from 6.5 to 8.5) in a 5-L fermentor using 2 M NaOH and 2 M H_2_SO_4_. As shown in Fig 2C and 2D, specific growth rate (μ) and specific acarbose production rate (*q*_*p*_) were estimated from experimental or fitted cell growth data (Fig 2A) and acarbose production (Fig 2B), respectively. Maintaining the pH at 7.0 resulted in a shorter lag time, increased DCW (21.65 g/L), and maximal μ (Fig 2A). Specific acarbose production increased with pH in the range 6.5 to 8.0, such that it was higher at pH 8.0 than at any other pH. At pH 8.0, acarbose yield was 4410 mg/L, a 31.8% increase over batch fermentation at the same pH.

As can be seen in Fig 2D, the highest *q*_p_ was obtained at pH 8.0. It should be noted that acarbose production was not accompanied by increased DCW. A similar result was observed by Lou et al. during β-carotene fermentation [12]. However, acarbose yield decreased when pH increased above 8.5. This was probably due to the negative influence on cell growth and acarbose synthesis caused by high pH (8.5). As shown in Fig 2E, reducing sugar content decreased significantly, from 20.2 g/L (0 h) to less than 6.0 g/L (168 h), which may have hampered acarbose biosynthesis.

It has been reported that medium pH affects enzyme synthesis, enzymatic processes, and transport across cell membranes [7,20,21]. Our results indicated that acarbose yield was optimal at a high pH (8.0). Therefore, maintaining a constant pH throughout the fermentation process was not suitable to achieve maximal acarbose production.

Biosynthetic studies have demonstrated that glutamate is the primary source of N-glycosidic bonds in acarbose [9]. Some reports have also shown that addition of ammonium to culture medium enhances the yield of streptomycin, neomycin, and gentamicin [22,23]. In addition, it was reported that ammonium was a good alternative primary nitrogen source under conditions of glutamate shortage, either by direct reductive amination of a biosynthetic intermediate or, more likely, by transfer of ammonium to α-ketoglutarate to form glutamate, in a reaction catalyzed by GDH/NADPH [24]. Under high culture medium pH, the stimulating effect of ammonium on acarbose formation may be due to the formation of ammonia from ammonium ions. Ammonia is generally toxic to microbes, as it is uncharged and can pass through cell membranes [25]. Our results suggested that an alkaline environment (pH 8.0) promoted acarbose production as a means to attenuate alkalinization of the cytoplasm.

### Effect of feeding glucose and maltose on acarbose production

According to the acarbose biosynthetic pathway, glucose and maltose can be directly incorporated into acarbose [26,27]. To explore the influence of these reducing sugars on *Streptomyces* M37 acarbose production, they were added at different concentrations (5–30 g/L) to the fermentation broth at 72 h, 96 h, 120 h, and 144 h. Addition of 20 g/L of reducing sugars led to an acarbose yield of 5160 mg/L, corresponding to a 17.0% increase with respect to the control (Table 2).

**Table 2:** Effect of the addition of reducing sugars (glucose and maltose) on *Streptomyces* M37 DCW and acarbose production at pH 8.0 in a 5-L fermentor.

Reducing sugar (g/L)^a^	DCW^b^ (g/L)	Acarbose titer (mg/L)
0	15.80 ± 0.76	4410 ± 210
5	15.98 ± 0.84	4525 ± 235
10	16.12 ± 0.71	4650 ± 240
15	16.23 ± 0.95	4832 ± 228
20	16.36 ± 0.86	5160 ± 253
25	16.43 ± 0.92	4920 ± 244
30	16.55 ± 0.89	4860 ± 230

^a^The mass ratio of maltose-to-glucose was 1:1.

^b^DCW was determined at 168 h.

Each value represents the mean of three separate determinations ± standard deviation.

To further define the optimal mass ratio of reducing sugars during acarbose production, we fed six different mixtures of glucose and maltose to the cultivation medium. During the fed-batch fermentation process in a 5-L fermentor at pH 8.0, 20 g/L of reducing sugars were fed at 72 h, 96 h, 120 h, and 144 h (an additional 5 g/L was added at each time point). According to the results in Table 3, acarbose biosynthesis was strongly affected by the ratio of glucose and maltose. When these were the sole carbon sources in the feeding medium, acarbose yield increased by 4.5% (*p* < 0.05) and 6.4% (*p* < 0.05) compared to batch fermentations, respectively. In the case of a 1:2 glucose-to-maltose ratio, an acarbose yield of 5472 mg/L was obtained, representing a 24.1% increase with respect to no glucose or maltose addition. Therefore, we concluded that a feed of 20 g/L with a 1:2 mass ratio of glucose-to-maltose favored acarbose production by *Streptomyces* M37.

**Table 3:** Effect of the maltose-to-glucose mass ratio in the feeding medium on acarbose production by *Streptomyces* M37 at pH 8.0 in a 5-L fermentor ^a^.

G:M^b^	DCW^c^ (g/L)	Acarbose titer (mg/L)
0:1	16.05 ± 0.82	4692 ± 214
1:1	16.22 ± 0.93	5162 ± 239
1:2	16.28 ± 0.88	5472 ± 246
1:3	16.11 ± 0.93	5271 ± 251
1:5	16.16 ± 0.86	5164 ± 263
1:0	16.29 ± 0.89	4608 ± 187
0:0	15.80 ± 0.94	4410 ± 168

^a^The total mass of glucose and maltose was 20 g/L.

^b^Mass ratio of glucose-to-maltose in the feeding medium.

^c^DCW was determined at168 h.

Each value represents the mean of three separate determinations ± standard deviation.

### Effect of a two-stage strategy on acarbose fermentation

Our results indicated that pH played an important role in cell growth and acarbose biosynthesis during the fermentation process. We investigated whether a two-stage cultivation strategy could maximize the yield of the target metabolite. Accordingly, optimal cell growth and acarbose production would be obtained by separately optimized conditions.

**Fig 3:**
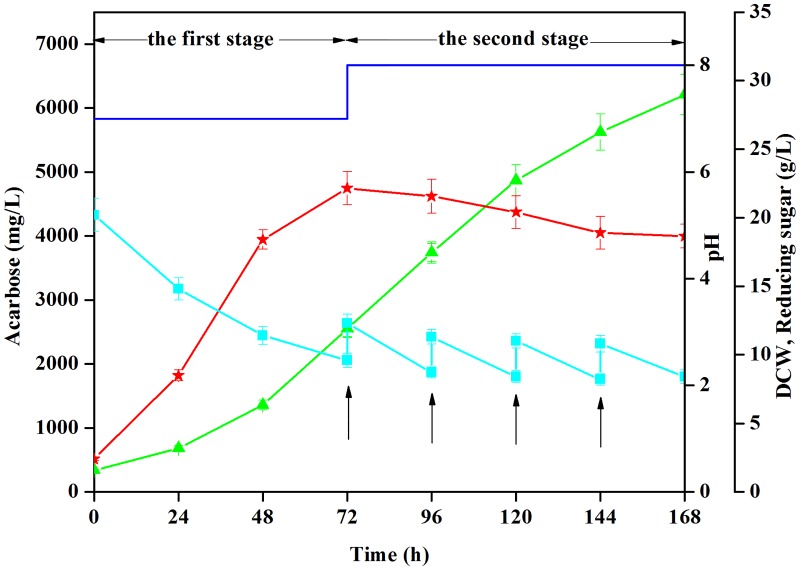
Effect of a two-stage fermentation strategy on acarbose production by *Streptomyces* M37. DCW (★), reducing sugars (■), and acarbose production (▲). Fermentations were carried out at 28°C and 200 rpm agitation speed. Glucose and maltose were fed at a 1:2 mass ratio and pH was adjusted with 2 M NaOH and 2 M H_2_SO_4_. Each value represents the mean of three separate determinations ± standard deviation (arrows indicate feeding times).

The following two-stage strategy was developed based on the foregoing results in a 5-L fermentor: pH was maintained at 7.0 during the first 72 h of fermentation to enhance cell growth; pH was then switched to 8.0 to facilitate acarbose biosynthesis. Meanwhile, 20 g/L of reducing sugars (1:2 mass ratio glucose-to-maltose) was added to the fermentation broth at 72 h, 96 h, 120 h, and 144 h (Fig 3). The effect of culture method on acarbose fermentation is summarized in Table 4. Using this strategy, the highest acarbose yield (6210 mg/L) was obtained at 168 h; this was 85.7% higher than that of traditional batch fermentation (initial pH 8.0).

**Table 4:** Effect of culture method on *Streptomyces* M37 acarbose fermentation in a 5-L fermentor.

pH	DCW* (g/L)	Acarbose titer (mg/L)	μ_max_ (1/h)
6.5 ^a^	20.54 ± 0.95	3206 ± 161	0.072 ± 0.004
7.0 ^a^	21.62 ± 1.06	3177 ± 157	0.075 ± 0.005
7.5 ^a^	19.40 ± 0.98	3223 ± 149	0.064 ± 0.004
8.0 ^a^	16.50 ± 0.95	3345 ± 146	0.053 ± 0.003
8.5 ^a^	15.20 ± 0.81	2470 ± 130	0.046 ± 0.004
7.0–8.0 ^b^	21.74 ± 1.05	6210 ± 286	0.076 ± 0.005

*Maximum DCW during the fermentation process.

^a^Constant pH control strategy.

^b^Two-stage pH control strategy, pH was maintained at 7.0 for the first 72 h and then switched to 8.0.

Each value represents the mean of three separate determinations ± standard deviation.

The obtained maximum acarbose titer yield was 27.3% higher than that reported by Wang et al. during fed-batch fermentation (4878 mg/L) [14]. Similarly, the observed final acarbose yield was 24.2% higher than the approximately 5000 mg/L obtained after 168 h of fermentation by Li et al. [11]. Multi-stage culture is a useful technology for the production of valuable metabolites [28–30]. The results of the present study suggested that the proposed two-stage control strategy effectively improved acarbose production.

### Effect of the two-stage strategy on G6PDH and GDH activity

G6PDH is the key enzyme in the HMP pathway. Studies on the biosynthesis of acarbose revealed that the cyclitol moieties of validamycin and acarbose originated from the HMP pathway, presumably via the intermediates sedoheptulose 7-phophate or ido-heptulose 7-phophate [8]. On the contrary, glutamate was reported to be the primary source of N-glycosidic bonds in acarbose. GDH is a widely distributed enzyme responsible for bridging carbon and nitrogen metabolism [9,31]; in addition, its physiological role may be anabolic and/or catabolic. The activities of G6PDH and GDH may affect acarbose formation by *Streptomyces* M37 (Fig 1).

**Fig 4:**
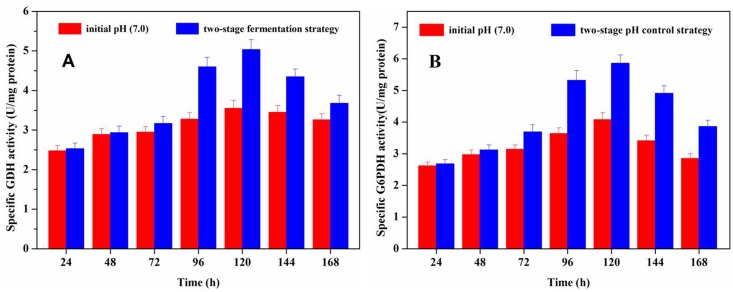
Specific activities of glutamate dehydrogenase (GDH) and glucose 6-phosphate dehydrogenase (G6DPH) during batch fermentation and the two-stage fermentation strategy. Each value represents the mean of three separate determinations ± standard deviation.

The effect of pH on G6PDH and GDH activity is shown in Fig 4. G6PDH and GDH exhibited higher enzymatic activity during the course of a two-stage pH control strategy than in traditional batch fermentation (initial pH 7.0). This indicates that in *Streptomyces* M37 these enzymes may promote glutamate production for subsequent acarbose synthesis. Our results indicated that both G6PDH and GDH activities were enhanced in cells cultivated using the two-stage strategy, which strongly correlated with acarbose production.

## Conclusions

In conclusion, we investigated the effect of pH and precursors (glucose and maltose) on acarbose biosynthesis by *Streptomyces* M37. Results indicate that a relatively low pH (7.0) is suitable for cell growth, whereas a high pH (8.0) favors acarbose synthesis. A two-stage fermentation strategy whereby pH was kept at 7.0 for the first 72 h and then shifted to 8.0 enhanced acarbose production. At the same time, glucose and maltose were fed in to promote acarbose accumulation. The specific activities of GDH and G6PDH were higher than in traditional batch fermentation (initial pH 7.0). The information obtained in this study may be valuable for large-scale production of acarbose.
